# A Spin Valve-Based Rhombus-Shaped Micro-Object Implementing a Full Wheatstone Bridge

**DOI:** 10.3390/s24020625

**Published:** 2024-01-18

**Authors:** Mikhail Milyaev, Larisa Naumova, Anastasiya Germizina, Tatyana Chernyshova, Anastasia Pavlova, Tatiana Krinitsina, Vyacheslav Proglyado, Vladimir Ustinov

**Affiliations:** M.N. Mikheev lnstitute of Metal Physics, Ural Branch, Russian Academy of Sciences, S. Kovalevskoi Street 18, Ekaterinburg 620990, Russia; naumova@imp.uran.ru (L.N.); germizina@imp.uran.ru (A.G.); chernyshova@imp.uran.ru (T.C.); anastasia.pavlova.1988@gmail.com (A.P.); krinitsina@imp.uran.ru (T.K.); proglyado@imp.uran.ru (V.P.); ustinov@imp.uran.ru (V.U.)

**Keywords:** spin valve, Wheatstone bridge, magnetic anisotropy, shape anisotropy, exchange bias field

## Abstract

Spin valves with a synthetic antiferromagnet were fabricated via magnetron sputtering. It was shown that the fabricated spin valve layers had a perfect microstructure and smooth interfaces, and therefore, an RKKY interaction dominated in the coupling of the ferromagnetic layers separated by a copper spacer. Rhombus-shaped micro-objects were fabricated from a single spin valve film. The thermomagnetic treatment procedure was found to form unidirectional anisotropy in the micro-object such that the values of the exchange bias fields in the rhombus’ nonparallel sides were opposite in sign. For the CoFeNi/Ru/CoFeNi synthetic antiferromagnet, we determined the differences between the ferromagnetic layer thicknesses at which the thermomagnetic treatment formed the same exchange bias all over each rhombus’ side. We also fabricated a sensor element in which each side of the rhombus was the shoulder of a Wheatstone bridge. After the thermomagnetic treatment procedure, each shoulder worked as an active magnetosensitive element, enabling the device to operate as a full Wheatstone bridge. The sensor output exhibited a step shape, high sensitivity to field changes, and significant magnetic hysteresis. Such characteristics are suitable for switching devices.

## 1. Introduction

Bridge circuit measurements have important advantages such as low measurement errors, high sensitivity, reductions in noise, and temperature drift [1,2,3,4]. A Wheatstone bridge electrical circuit consists of two parallel branches, each consisting of two arms containing a resistor element. If a Wheatstone bridge is used in magnetic field sensors, then elements, the electrical resistivity of which depend on the applied magnetic field, are used as resistors in each arm. The supply voltage *U*_in_ is applied to the parallel branches, and the potential difference *U*_out_ between the middle points of the branches is measured. The maximum value of *U*_out_/*U*_in_ can be achieved if the resistance increases in two bridge arms and decreases in two other arms with a change in the magnetic field. In this case, all four sensitive elements contribute to the output signal, and the circuit is a full bridge. In microelectronics, a spin valve (SV) nanostructure with giant magnetoresistance (GMR) is often used as a magnetosensitive material [5,6,7].

An SV consists of two ferromagnetic (FM) layers, the free layer (FL) and the pinned layer (PL), which are separated by a nonmagnetic spacer, usually made from Cu. The magnetic moment (**M**_p_) of the pinned layer is fixed by the exchange interaction with the adjacent antiferromagnetic (AF) layer [8]. In this way, the magnetic reversal hysteresis loop for the PL is shifted. The high-field loop shift (*H*_ex_) depends on the interaction between the AF and PL. For the FL, the field shift of the hysteresis loop from *H* = 0 (*H*_j_) is small. In the fields |*H*_j_| < |*H*| < |*H*_ex_|, the magnetic moments of the FL and PL are opposite, and there is a plateau of the magnetoresistive curve corresponding to the maximum magnetoresistance (*MR*_max_). *H*_j_ depends on the FM layers’ interaction through the nonmagnetic spacer. These interactions are the result of competition between the dipolar ferromagnetic and oscillating RKKY exchange interactions [9]. If dipolar ferromagnetic interactions are dominant, then *H*_j_ and *MR*_max_ decrease with an increase in spacer thickness. If the standard deviation of the interlayer roughness is no more than 0.3–0.5 nm [10,11], then the RKKY interactions can be dominant. In this case, *H*j changes periodically with an increase in the spacer thickness, enabling an SV with *H*j ≈ 0 and a large *MR*_max_ to be obtained [12].

The pinned layer in an SV can be replaced by a synthetic antiferromagnet (SAF) to reduce the magnetostatic interactions between the FM layers and increase the operating temperature range. An SAF consists of two FM layers coupled through a layer of Ru [13,14,15]. The thickness of the Ru layer (0.7–0.9 nm) corresponds to the maximum antiferromagnetic exchange interaction [16]. In this case, the FM layer adjacent to the AF layer is called the pinned layer (PL), and the second FM layer in the SAF is called the reference layer (RL).

The magnetic and magnetoresistive properties of an SV have several types of anisotropy: (1) uniaxial easy axis (EA) anisotropy, induced during deposition in a magnetic field; (2) unidirectional anisotropy with the pinning direction (PD), arising due to the interaction between the pinned and AF layers; and (3) shape anisotropy in micro-objects. The PD can be changed via thermomagnetic treatment (TMT) [1].

It is difficult to implement a full Wheatstone bridge during magnetic field microsensor fabrication from a single SV film because the resistance (*R*_n_, n = 1, 2, 3, 4) of all the bridge arms changes equally with a change in the magnetic field, and all the d*R*_n_/d*H* values are the same in sign. d*R*_1,3_/d*H* and d*R*_2,4_/d*H* should be opposite in sign to contribute to the output voltage *U*_out_ from all the bridge elements. This result is possible for the SVs in the Wheatstone bridge if their PDs are mutually opposite. The following methods are used to obtain this result: the two-stage deposition of SVs with different PDs [1] and EAs [17] or with different compositions [18,19] onto the corresponding parts of the substrate; TMT in the field corresponding to the spin-flop state in the SAF [16,17]; and the use of a permanent magnet to create additional oppositely directed field components in the active elements of the bridge [20].

In this work, spin valves with predominant RKKY interactions between the free and reference layers are studied. The formation of an exchange shift in the pinned layer under the combination of shape anisotropy and uniaxial anisotropy is also investigated. A thermomagnetic treatment mode to form an opposite exchange shift in the nonparallel sides of a rhombus-shaped micro-object was found, and the full Wheatstone bridge circuit was realized.

## 2. Materials and Methods

SVs with the composition Ta(5)/[Ni_80_Fe_20_]_60_Cr_40_(5)/Co_70_Fe_20_Ni_10_(*t*_FL_)/Cu(*t*_Cu_)/Co_70_Fe_20_Ni_10_(*t*_RL_)/Ru(0.8)/Co_70_Fe_20_Ni_10_(*t*_PL_)/Fe_50_Mn_50_(10)/Ta(5) were deposited on glass substrates via dc magnetron sputtering in a magnetic field of 80 Oe and applied in a film plane. Layer thicknesses are given in nm. A computer program controlled the process of deposition for the multilayer nanostructure. This program set the sequence of deposition of the layers, the time of deposition for each layer, the time of launching argon gas into the chamber, the power of the magnetrons, argon pressure, substrate temperature, and substrate rotation speed. The base pressure of the residual gasses in the sputtering chamber was less than 5 × 10^−7^ Pa. Sputtering was carried out at room temperature under an argon pressure of 0.1 Pa and magnetron power of 100 W. The nanolayer thickness was nominal and calculated from the measured deposition rate with a pre-assigned sputtering time. The applied experimental procedure allowed us to change the layer deposition time with a step of 0.1 s (corresponding to a step of 0.01 nm) at a copper deposition rate of 7 nm/min and a deposition rate of 2.8–3 nm/min for the rest of the layers. The materials’ deposition rates were determined by making calibration films and measuring their thickness values using a Zygo NewView 7300 (Zygo, Middlefield, OH, USA) white light interferometer with an accuracy of ±0.02 nm at full film thickness (40–60) nm. The thicknesses of the Cu and FM layers were varied, and Co_70_Fe_20_Ni_10_ was used as the FM layer. For this alloy, saturation magnetization was 13% higher and coercivity was 4.5 times lower than the values for Co_90_Fe_10_ commonly used in SVs [21]. The buffer layer Ta/[Ni_80_Fe_20_]_60_Cr_40_ promoted the formation of the <111> texture [22,23] and a decrease in crystallite size, as well as the interlayer roughness of the nanostructured film [24,25].

Analyses of the materials’ microstructure were carried out via X-ray diffractometry on a DRON-3M (Bourevestnik, St. Petersburg, Russia) diffractometer under CoKα_1_ radiation (λ = 1.7889 Å) and via transmission electron microscopy on a Tecnai G-30 (FEI, Hillsboro, OR, USA). A special technique was used to produce TEM samples (foil). The sample on a glass substrate was thinned using sandpaper, and then ion etching was carried out using a PIPS II 695 (GATAN, Pleasanton, CA, USA).

The magnetic microstructures of the micro-objects were investigated by using a scanning probe microscope Solver Next (NT-MDT, Zelenograd, Moscow, Russia) in the magnetic force microscopy (MFM) mode.

Magnetoresistive measurements and TMT were carried out in the setup based on a Bruker electromagnet and a LakeShore 336 temperature controller (Lake Shore Cryotronics, Westerville, OH, USA). TMT involved heating in a helium atmosphere to a temperature *T*_TMT_ = 448 K, which exceeded the blocking temperature *T*_b_ = 433 K for SVs based on an antiferromagnetic FeMn alloy [26]. The magnetoresistance was determined as *MR =* (*R*(*H*) − *R*_s_)/*R*_s_, where *R*(*H*)—resistance of the sample in a magnetic field, and *R*_s_—resistance of the sample in a saturation field. The error of the relative resistance value was 0.05%. These experimental results were all obtained for the “Current In the Plane of the layers” (CIP) geometry.

**Figure 1:**
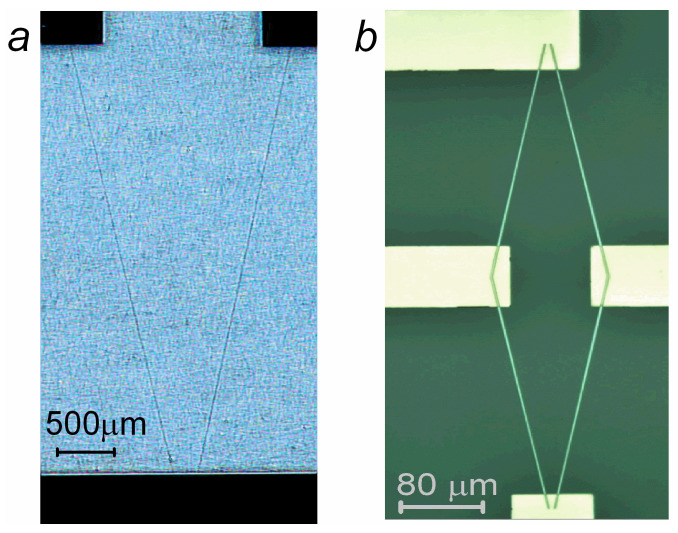
Images of V-shaped micro-object made from 2 microstrips (**a**) and rhombus-shaped micro-object made from 4 microstrips (**b**) with contact pads.

Micro-objects were fabricated via laser lithography on DWL 66+ (Heidelberg Instruments Mikrotechnik GmbH, Heidelberg, Germany) and reactive ion etching on a PlasmaPro 80 RIE (Oxford Instruments, Abingdon-on-Thames, UK). The contact pads were fabricated using the lift-off procedure. Two types of micro-objects (Figure 1) were prepared. (1) V-shaped micro-objects were made from two microstrips forming a corner with Cu contact pads at the apex of the corner and ends of the microstrips. The corner angle (α) was 20 or 40°, and its bisector coincided with the EA. (2) Rhombus-shaped micro-objects included 315 μm long and 2 μm wide microstrips and Cu pads at the rhombus vertices. The acute angles were α = 20 or 40°, and the long rhombus diagonal coincided with the EA.

## 3. Results

### 3.1. Microstructural Studies

**Figure 2:**
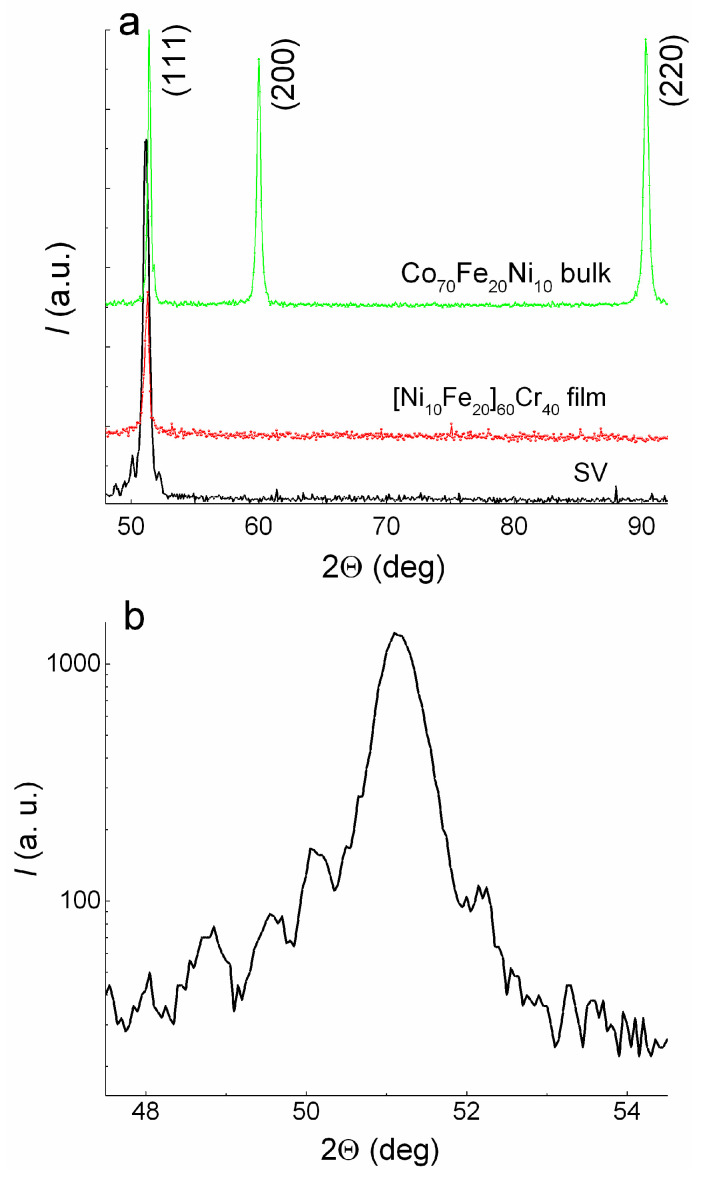
(**a**) X-ray diffraction patterns for the SV of the Ta(5)/NiFeCr(5)/CoFeNi(3.5)/Cu(3.3)/CoFeNi(3.5)/Ru(0.8)/CoFeNi(3)/FeMn(10)/Ta(5) composition (black line), NiFeCr film with a thickness of 60 nm (red line), and bulk sample of the CoFeNi alloy (green line). (**b**) Thickness oscillations (satellites) around the (111) peak of the SV diffraction pattern.

The Co_70_Fe_20_Ni_10_ alloy, [Ni_80_Fe_20_]_60_Cr_40_ alloy, and Cu and Fe_50_Mn_50_ antiferromagnetic alloy have the same cubic face-centered (fcc) crystal structure and similar lattice parameter values. Figure 2a shows the coincidence of the angular position of the (111) peak for the bulk Co_70_Fe_20_Ni_10_ alloy, the thin film of the [Ni_80_Fe_20_]_60_Cr_40_ alloy, and the SV containing the layers of these alloys. A <111> texture can be seen in the film and SV, with no other peaks in the fcc structure. The full width at the half-maximum of the rocking curve (ω-scan) for the (111) peak of SV is 4.4 degrees.

Thickness oscillations (satellites) are clearly visible around the (111) peak (Figure 2b) in the SV X-ray diffraction patterns. The presence of satellites shows that the SV films are of high quality and have smooth interfaces. Such satellites appear when X-ray radiation interferes on a layer with a uniform microstructure and smooth boundaries. The thickness (*d*) of such a layer can be estimated from the period of the oscillations with Selyakov’s formula [27,28]:

(1) $$d=\frac{\lambda }{\Delta \left(2\Theta \right)cos\Theta_{B}}$$

where *λ* is the X-ray wavelength, *Θ_B_* is the Bragg angle for the (111) peak, and Δ(2*Θ*) is the period of the oscillations. The value of *d* determined from formula (1) coincides with an accuracy of 0.1 nm for the total nominal thickness of the NiFeCr(5)/CoFeNi(3.5)/Cu (3.3)/CoFeNi(3.5) layers in the spin valve. Thus, in this part of the SV, the microstructure is uniform, and the microstructure of the layers become aligned.

**Figure 3:**
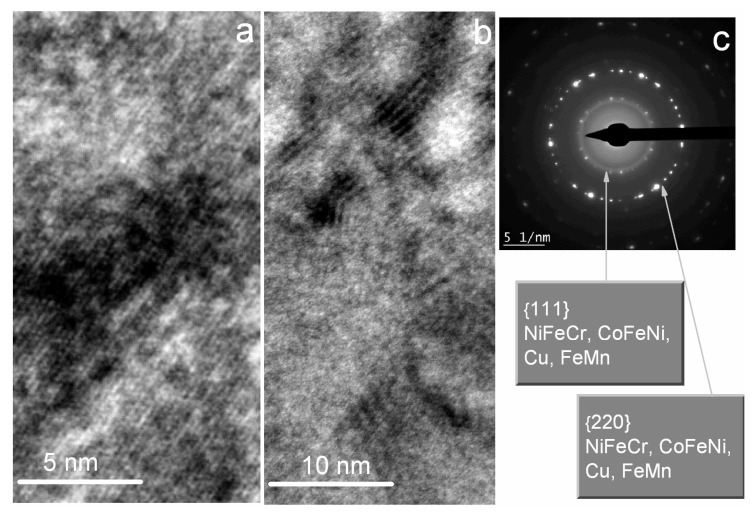
TEM high-resolution images (**a**,**b**) and electron diffraction pattern (**c**) for the SV of the Ta(5)/NiFeCr(5)/CoFeNi(3.5)/Cu(3.3)/CoFeNi(3.5)/Ru(0.8)/CoFeNi(3)/FeMn(10)/Ta(5) composition.

Figure 3 shows the results of the TEM investigation. In the electron diffraction pattern, Debye rings for NiFeCr, CoFeNi, Cu, and FeMn are common because they have the same fcc structure and similar lattice parameters. Note that the {220} ring is the brightest, while the {111} ring is weak. It is typical for the <111> texture and consistent with the results of the X-ray diffraction study. High-resolution images show thin parallel bands (Figure 3a). These are a direct resolution of the atomic plane projections on the image plane. Figure 3b shows the moiré lines. These lines are a result of the interference of electron beams diffracted by crystal lattices in adjacent layers. In multilayer structures parallel to each other, moiré lines appear if the lattice mismatch of the layers is low, and the number of dislocations and packaging defects is small.

The studied SVs featured layers with a perfect microstructure and low lattice mismatch along with an excellent <111> texture. Such properties of the microstructure were obtained by using the layer materials with similar crystal structures and the buffer layer of the Ta(5)/NiFeCr(5) composition. In [12,24], a similar high-quality microstructure was achieved in the superlattices and spin valves sputtered onto the Ta/NiFeCr buffer layer. In the present investigation, such microstructural properties are necessary to obtain smooth interfaces and a high prevalence of RKKY interlayer coupling.

### 3.2. Exchange Coupling of Free and Reference Layers

**Figure 4:**
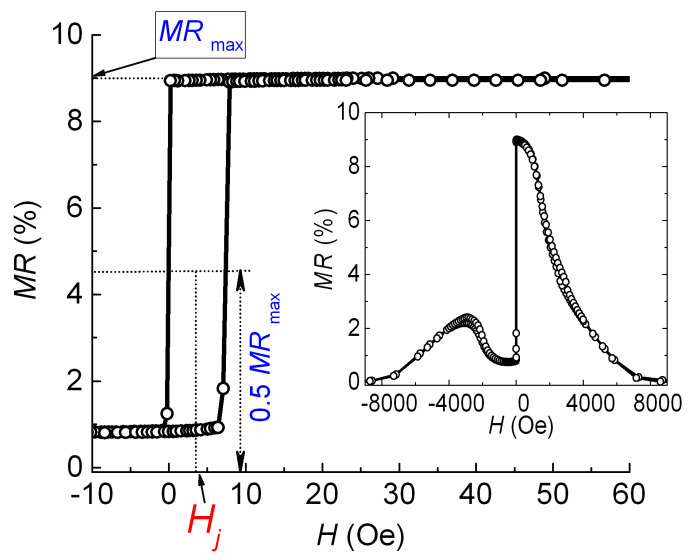
Low field part of the magnetoresistive curve for the SV of the Ta(5)/NiFeCr(5)/CoFeNi(5.5)/Cu(2.2)/CoFeNi(2.3)/Ru(0.8)/CoFeNi(2)/FeMn(10)/Ta(5) composition. The inset shows the *MR*(*H*) curve in a wide range of fields.

Magnetoresistive curves were measured for SVs with *t*_Cu_ = 1.8 ÷ 2.4 nm in the field applied parallel to PD || EA. Figure 4 shows the *MR*(*H*) curve for the SV with *t*_Cu_ = 2.2 nm. Figure 4 shows how the values of *MR*_max_ and the shift of low field hysteresis loop (*H*_j_) were estimated. For spin valves with weak interactions between the free and pinning layers, the low field loop retains a rectangular shape in the case of small values for the angle of deviation PD from the EA [9,29].

**Figure 5:**
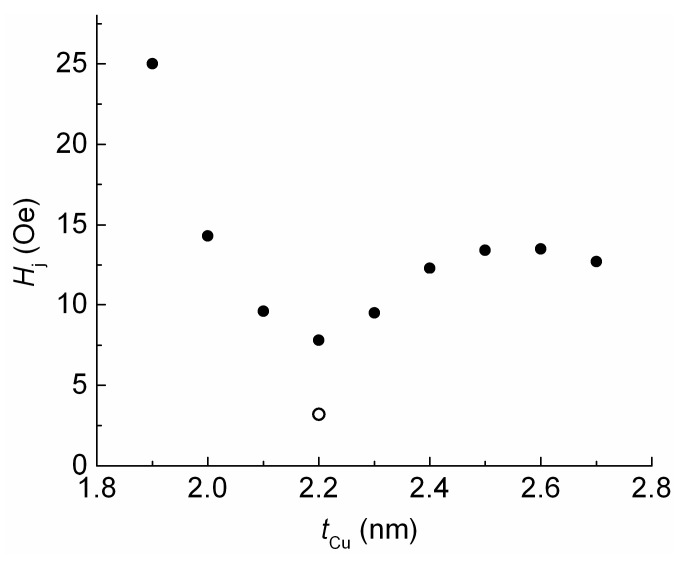
The dependence of the low field hysteresis loop shift on the thickness of the Cu layer for SVs of the Ta(5)/NiFeCr(5)/CoFeNi(4)/Cu(*t*_Cu_)/CoFeNi(4)/Ru(0.8)/CoFeNi(3.5)/FeMn(10)/Ta(5) composition (solid symbols) and the shift in the low field hysteresis loop for the SV of the Ta(5)/NiFeCr(5)/CoFeNi(5.5)/Cu(2.2)/CoFeNi(2.3)/Ru(0.8)/CoFeNi(2)/FeMn(10)/Ta(5) composition (open symbol).

The *H*_j_ value depends on the interaction between the free and reference FM layers. Ordinarily, if ferromagnetic dipolar coupling dominates, *H*_j_(*t*_Cu_) dependence decreases monotonously. In our case, *H*_j_(*t*_Cu_) dependence has an oscillating behavior. Thus, we may conclude that RKKY interlayer coupling dominates (Figure 5).

**Figure 6:**
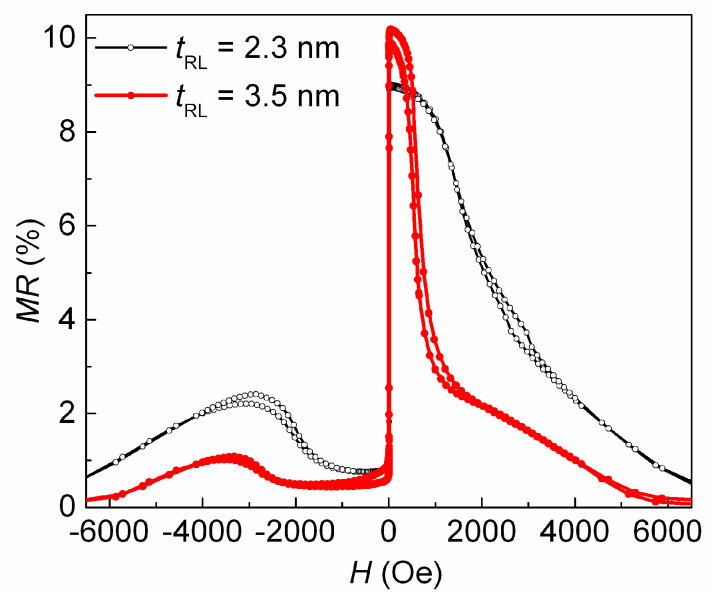
Magnetoresistive curves for SVs of the Ta(5)/NiFeCr(5)/CoFeNi(5.5)/Cu(2.2)/CoFeNi(*t*_RL_)/Ru(0.8)/CoFeNi(2)/FeMn(10)/Ta(5) composition for *t*_RL_ = 2.3 and 3.5 nm.

**Figure 7:**
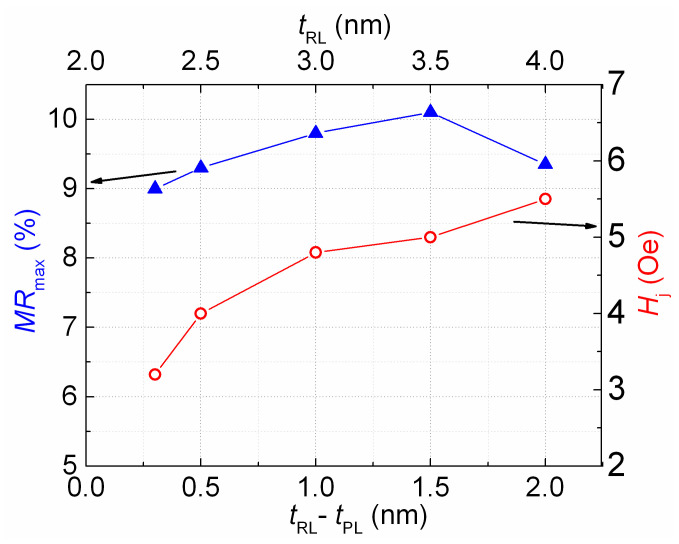
Dependencies of the low field hysteresis loop shift (red line and circles, shift values are given on the right axis) and the maximal magnetoresistance (blue line and triangles, magnetoresistance values are given on the left axis) on the reference layer thickness for the SVs of the Ta(5)/NiFeCr(5)/CoFeNi(5.5)/Cu(2.2)/CoFeNi(*t*_RL_)/Ru(0.8)/CoFeNi(2)/FeMn(10)/Ta(5) composition.

The minimum value of *H*_j_ was obtained at *t*_Cu_ = 2.2 nm. Changing the thickness of the ferromagnetic layers in the SV is also an effective way to influence the amount of interlayer interaction [30]. Thus, we changed the thickness of the FM layers to increase *MR*_max_ and further reduce *H*_j_. We achieved an *H*_j_ value close to *H* = 0 (open symbol in Figure 5) and *MR*_max_ = 9% at a *t*_FL_ of 5.5 nm and *t*_PL_ of 2 nm. In the SV of the obtained composition, we changed the reference layer thickness (*t*_RL_). Thus, the difference (*t*_RL_ − *t*_PL_) between the FM layers in the SAF and the total magnetic moment of the SAF changed. Figure 6 and Figure 7 show the changes in the magnetoresistive curves and the values of *H*_j_ and *MR*_max_ that occurred due to increasing the value of *t*_RL_.

When the thickness and, accordingly, the magnetic moment of the reference layer increase, the interlayer coupling and *H*_j_ value naturally increase. The value of *MR*_max_ also varies slightly.

Further analyses will be carried out on this series of SVs with a small shift in the low field hysteresis loop and different values of the reference layer magnetic moment.

### 3.3. Change in the Pinning Direction in Spin Valve Films and Micro-Objects during Thermomagnetic Treatment

**Figure 8:**
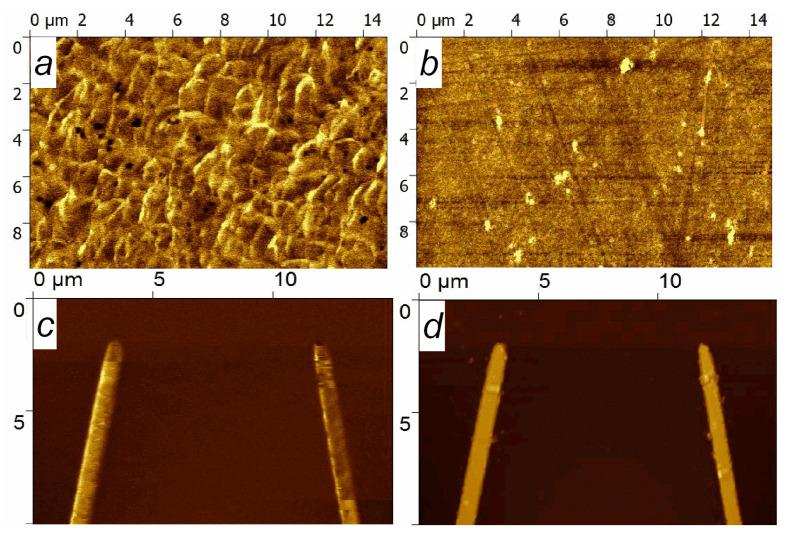
Topography (**b**,**d**) and MFM (**a**,**c**) images obtained for the film (**a**,**b**) and the V-shaped micro-object (**c**,**d**).

The thermomagnetic treatment procedure includes annealing at *T*_TMT_ and subsequent cooling in the applied magnetic field *H*_TMT_. If *T*_TMT_ > *T*_b_, the exchange interaction at the boundary of CoFeNi/FeMn is destroyed. Then, the initial unidirectional anisotropy and initial PD in the SV disappear. Cooling in the applied magnetic field then forms new unidirectional anisotropy and new PD1. The direction of PD1 coincides with the direction of the magnetic moment of the adjacent FM layer [31,32]. We performed two consecutive TMT procedures for the following objects: the V-shaped SV micro-object and the SV film. We performed the first TMT(1) with *H*_TMT_ = 9 kOe exceeding the magnetic saturation field and the second TMT(2) with *H*_TMT_ ≈ 0. *H*_TMT_ was always directed perpendicular to the EA. After TMT(2), we investigated the magnetic structure of the V-shaped micro-object and SV film using magnetic force microscopy. Figure 8 shows the topography and corresponding MFM images obtained for the film and the V-shaped micro-object.

The MFM image of the film shows an irregular magnetic structure. The majority of each strip has one magnetic order with small areas of a different magnetic order. Magnetic contrast shows that the predominant magnetic order differs between the strips. In the MFM image (Figure 8c), the left strip is lighter than the right strip, while there is no such difference in the topographic image. Thus, after the same TMT, different magnetic structures were formed in the film and the V-shaped micro-object. The presumed reason for the observed differences is as follows.

**Figure 9:**
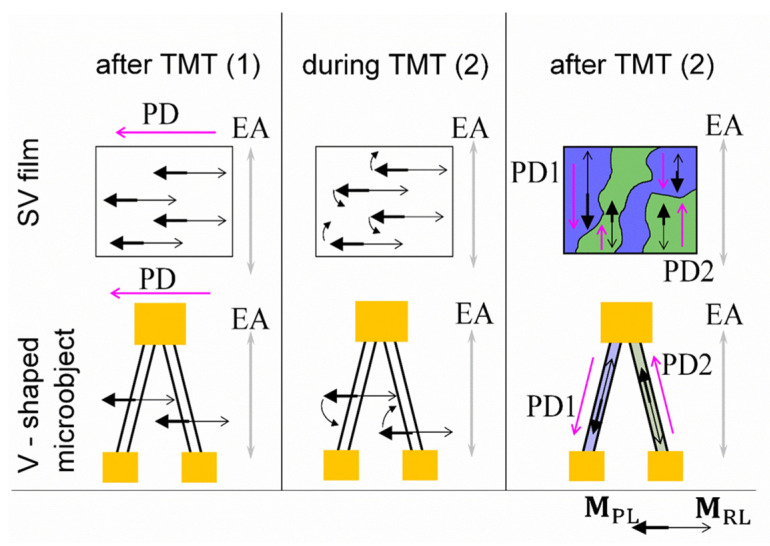
Schematic representation of the turn of reference (thin black arrow) and pinned (thick black arrow) layer magnetic moments in the SV film and SV micro-object during TMT. PD (pink arrow) changes and EA direction (grey arrow) is unchanged during TMT. Green and blue colors correspond to areas with different PDs.

Here, the magnetic moments of the pinned and reference layer (**M**_PL_ and **M**_RL_) are antiferromagnetically coupled and opposite to each other in *H*_TMT_ ≈ 0. In the SV film during TMT(2) at *T* = *T*_TMT,_ the uniaxial anisotropy controls the turn of **M**_PL_ and **M**_RL_; thus, clockwise and counterclockwise turns are equally probable (Figure 9). During subsequent cooling, the exchange interaction in the CoFeNi/FeMn boundary fixes this different magnetic ordering and forms the new pinning directions PD1 and PD2 in different regions of the film. PD1 and PD2 are antiparallel and collinear with EA. In the micro-objects, the shape anisotropy competes with the uniaxial anisotropy. If the shape anisotropy dominates, then it controls the turn of **M**_PL_ and **M**_RL_ during TMT(2) at *T* = *T*_TMT_. With our experimental geometry (Figure 9), in one strip of the V-shaped micro-object, **M**_PL_ and **M**_RL_ turn clockwise, and in the other strip, they turn counterclockwise. Thus, after cooling, the new pinning directions PD1and PD2 are collinear with the strips. If the angle between the strips is 20 or 40°, the angle between PD1 and PD2 will be 160 or 140°, respectively.

**Figure 10:**
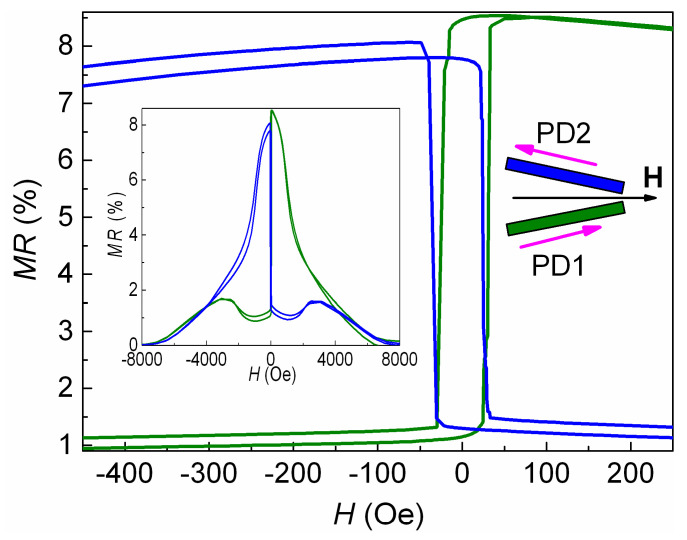
Low field parts of the magnetoresistive curves measured for each strip of the V-shaped SV micro-object. The applied magnetic field direction with respect to PD1 and PD2 is shown in the insert on the right. The inset on the left shows the *MR*(*H*) curves in a wide range of fields. The color of the curve corresponds to the color of strip.

Magnetoresistive curves measured for each strip of the V-shaped micro-object in the magnetic field applied parallel to EA are shown in Figure 10. The values of *H*_ex_ for two different strips are opposite in sign. This result is in good agreement with the fact that in one strip, the projection of PD onto the applied field is positive, and in the other, the projection of PD onto the applied field is negative.

In the low fields, d*MR*/d*H* > 0 for the green magnetoresistive curve, and d*MR*/d*H* < 0 for the blue magnetoresistive curve. Note that this PD arrangement was obtained in different strips of a single micro-object under two TMTs in a direction-fixed magnetic field.

### 3.4. Full Wheatstone Bridge Based on Rhombus-Shaped Spin Valve Micro-Object

**Figure 11:**
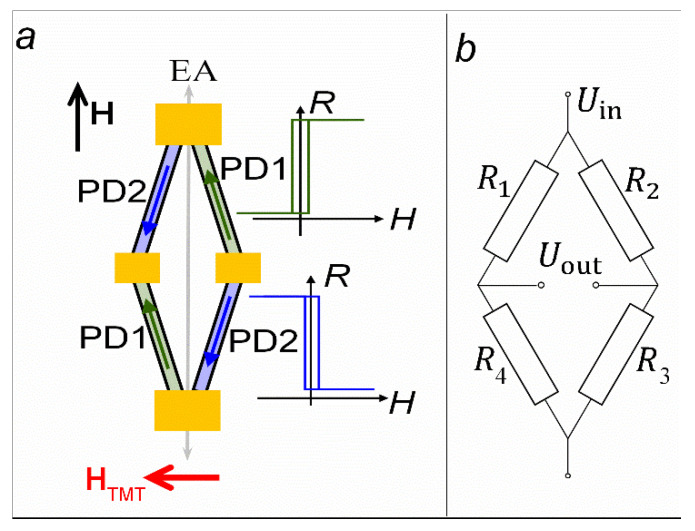
Scheme of PD arrangement in the rhombus-shaped micro-object sides after TMT(1) and TMT(2) (**a**) and the electrical circuit of the Wheatstone bridge (**b**). The inset in (**a**) shows *R*(*H*) dependencies for *R*_1_, *R*_3_ (blue) and *R*_2_, *R*_4_ (green).

TMT(1) and TMT(2) procedures were performed for the rhombus-shaped spin valve micro-object. Figure 11 shows the PD arrangement with rhombus sides, which can be expected if one considers the rhombus as two V-shaped micro-objects. This mutual PD arrangement is required to implement the Wheatstone bridge, in which d*R*_1,3_/d*H* and d*R*_2,4_/d*H* are opposite in sign. Thus, each SV element makes an active contribution to the output signal.

**Figure 12:**
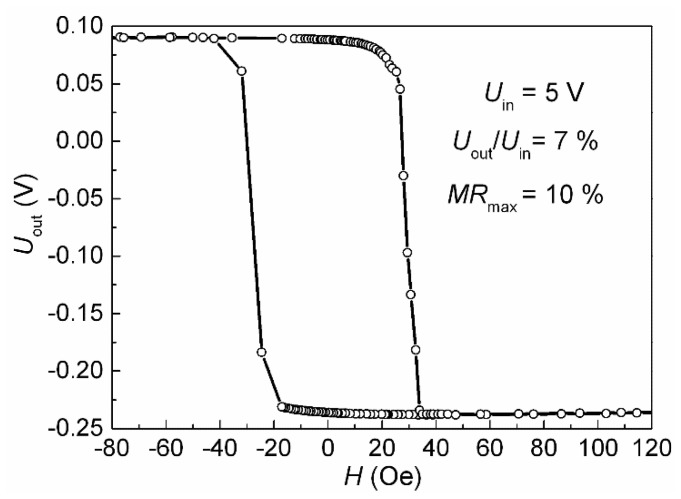
Wheatstone bridge output under the magnetic field sweep along EA for the rhombus-shaped micro-object based on the SV of the Ta(5)/NiFeCr(5)/CoFeNi(5.5)/Cu(2.2)/CoFeNi(3.5)/Ru(0.8)/CoFeNi(2)/FeMn(10)/Ta(5) composition.

We applied the supply voltage (*U*_in_) to the long diagonal of the rhombus and measured output voltage (*U*_out_) in the short diagonal of the rhombus under the magnetic field swept along the EA (Figure 12).

The maximal output of the full Wheatstone bridge was estimated from *U*_out_/*U*_in_ = Δ*R*/*R*, where *R* is the resistance of each of the four elements, and Δ*R* is the resistance change in the applied magnetic field. For the half Wheatstone bridge with two active sensing elements, the maximal output was *U*_out_/*U*_in_ = 0.5Δ*R*/*R* [33]. Thus, for the full Wheatstone bridge with four active SV elements, we obtained *U*_out_/*U*_in_ = *MR*_max_, where *MR*_max_ is the maximal magnetoresistance of each SV element. In our case, *U*_out_/*U*_in_ = 7%, while *MR*_max_ = 10% (Figure 12 and Figure 6, respectively). Thus, 0.5*MR*_max_ < *U*_out_/*U*_in_ < *MR*_max_. A possible reason for this result could be the presence of small areas of undesirable PD orientation in the rhombus-forming strips. These areas are visible in the image of the magnetic structure (Figure 8c) in the upper part of the left strip and in the lower part of the right strip.

### 3.5. Formation of Opposite Pinning Directions in Rhombus-Shaped Micro-Objects Based on Spin Valves with Different Thicknesses of the Reference Layer

An increase in *t*_RL_ led to an increase in the total effective magnetic moment (**M**_eff_) of antiferromagnetically coupled **M**_PL_ and **M**_RL_, with *M*_eff_ = *M*_RL_ − *M*_PL_. During TMT(2) at *T* > *T*_b_, the magnetic moments were arranged in a way that minimized the anisotropy anisotropic energy. We estimated the shape anisotropy field using the research in [34], in which the demagnetizing factors of the general ellipsoid were reported. For the long strip, the demagnetizing factor was approximately *t*/*w*, where *t* is the thickness of the FM layer and *w* is the width of the strip. Hence, the anisotropy field was calculated as the sum of two terms: *H*_a_ = 2*K*_u_/*M*_eff_ + 4 *M*_eff_ *t*/*w*, where *K*_u_ is the uniaxial anisotropy constant, and *t* = *t*_RL_ + *t*_PL_. We changed the relationship between the two terms of the anisotropy field by changing *t*_RL_. In this way, we changed the magnetic moments’ arrangement after TMT(2).

**Figure 13:**
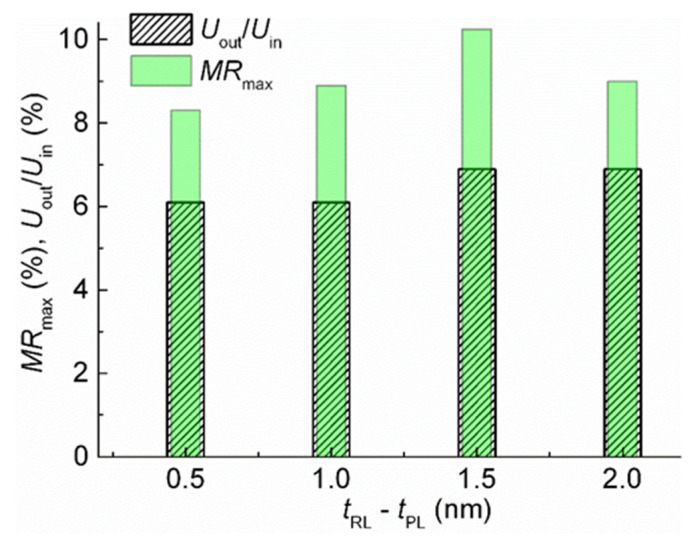
The dependencies of the Wheatstone bridge *U*_out_/*U*_in_ ratio and SV strip *MR*_max_ value on the thickness of the reference layer for rhombus-shaped micro-objects with α = 40°.

**Figure 14:**
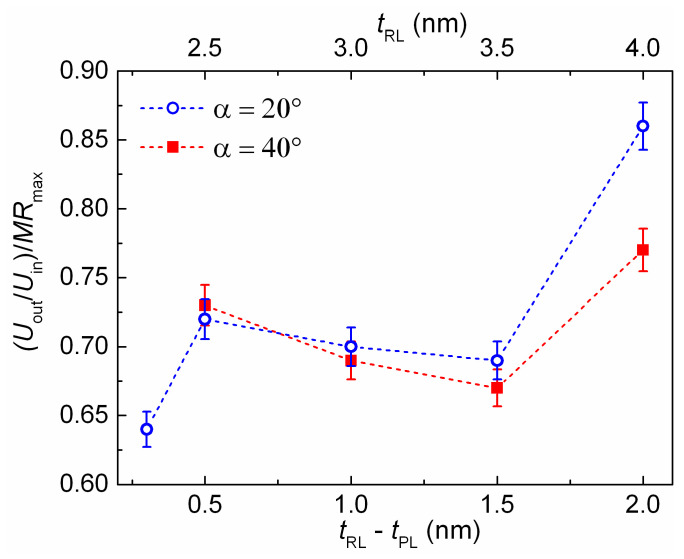
The dependencies of the relation between *U*_out_/*U*_in_ and *MR*_max_ on the thickness of the reference layer for rhombus-shaped micro-objects with α = 20 and 40°.

Rhombus-shaped micro-objects with an angle α = 20 and 40° were fabricated from films of SVs with *t*_RL_ = 2.3, 2.5, 3.0, 3.5, and 4 nm. TMT(1) and TMT(2) were performed consistently. Then, the Wheatstone bridge output voltage was measured in the short diagonal of the rhombus under a magnetic field swept along the EA. Figure 13 shows bar charts to compare the Wheatstone bridge *U*_out_/*U*_in_ ratio with the *MR*_max_ value of the SV strips. For convenience of comparison, we also plotted the relation between *U*_out_/*U*_in_ and *MR*_max_ as a function of the reference layer thickness (Figure 14).

For rhombus-shaped micro-objects with α = 20°, the minimal difference between *U*_out_/*U*_in_ and *MR*_max_ was found for samples with the maximum *t*_RL_ and maximum difference in FM layer thicknesses in the SAF. The effect of shape anisotropy on the alignment of the magnetic moments under TMT likely increases with an increase in the total magnetic moment of the antiferromagnetically coupled M_PL_ and M_RL_. For rhombus-shaped micro-objects with α = 40°, the difference between *U*_out_/*U*_in_ and *MR*_max_ is practically the same under maximal and minimal *t*_RL_. This result could be caused by different effects of uniaxial anisotropy due to the different micro-object geometries. When the angle α changes from 20 to 40°, the deviation of the strip from EA increases. As *M*_eff_ increases, coupled M_PL_ and M_RL_ can be set not only along the strip but also along EA in some areas of the strip.

## 4. Discussion

The proposed method for implementing a full Wheatstone bridge scheme with SVs as magnetically sensitive materials is simple. This method does not require the separate sputtering of the SV elements with an exchange bias that is opposite in sign or a separate TMT for each SV element. Nevertheless, there are a number of requirements for the successful application of this method. To prevent accidental splitting into magnetic domains, the microstructure of the layers should contain minimal defects, and the interlayer boundaries should be smooth. The SV elements with opposite exchange bias should change their magnetoresistance under the same low fields. Therefore, the shift of the low field hysteresis loop should be close to *H* = 0. To increase the ratio between *U*_out_/*U*_in_ and *MR*_max_ for each SV element, it is necessary to increase the difference in the thicknesses of the reference and pinned layers and, accordingly, the total magnetic moment of the SAF. The proposed TMT procedure is also effective for SVs based on antiferromagnetic alloys with higher Neel temperatures and, accordingly, higher *T*_b_ values [35]. In this case, it is necessary to use a higher temperature *T*_TMT_ > *T*_b_.

## 5. Conclusions

The results showed that shape anisotropy controls the turn of magnetic moments in spin valve micro-objects under the low magnetic fields.

We used the TMT procedure, which makes it possible to obtain exchange bias fields that are opposite in sign and a nearly collinear arrangement of the axes of uniaxial and unidirectional anisotropy in separate elements of the Wheatstone bridge circuit. The procedure includes two subsequent TMTs in the applied magnetic field perpendicular to the easy axis. During the first TMT, the field exceeded the saturation field, and during the second TMT, the field was close to *H* = 0. The formation of the opposite exchange bias in non-parallel sides of the rhombus-shaped micro-object was caused by the predominance of shape anisotropy in magnetic reversal and during TMT. The output signal of the complete Wheatstone bridge sensor had a step-like shape. Such characteristics are in demand for switching devices.

## Data Availability

The original contributions presented in the study are included in the article, further inquiries can be directed to the corresponding author.
